# Hybrid integral transform analysis of supercooled droplets solidification

**DOI:** 10.1098/rspa.2020.0874

**Published:** 2021-04

**Authors:** Igor S. Carvalho, Renato M. Cotta, Carolina P. Naveira-Cotta, Manish K. Tiwari

**Affiliations:** ^1^ Petrobras S.A., Rio de Janeiro, Brazil; ^2^ Laboratory of Nano and Microfluidics and Microsystems, LabMEMS, Mechanical Engineering Department, POLI and COPPE, UFRJ, Federal University of Rio de Janeiro, Brazil; ^3^ General Directorate of Nuclear and Technological Development, DGDNTM, Brazilian Navy, Rio de Janeiro, Brazil; ^4^ Nanoengineered Systems Laboratory, UCL Mechanical Engineering, University College London, London WC1E 7JE, UK; ^5^ Wellcome/EPSRC Centre for Interventional and Surgical Sciences (WEISS), University College London, London W1 W 7TS, UK

**Keywords:** integral transforms, GITT, moving boundary, supercooled droplet, icing

## Abstract

The freezing phenomena in supercooled liquid droplets are important for many engineering applications. For instance, a theoretical model of this phenomenon can offer insights for tailoring surface coatings and for achieving icephobicity to reduce ice adhesion and accretion. In this work, a mathematical model and hybrid numerical–analytical solutions are developed for the freezing of a supercooled droplet immersed in a cold air stream, subjected to the three main transport phenomena at the interface between the droplet and the surroundings: convective heat transfer, convective mass transfer and thermal radiation. Error-controlled hybrid solutions are obtained through the extension of the generalized integral transform technique to the transient partial differential formulation of this moving boundary heat transfer problem. The nonlinear boundary condition for the interface temperature is directly accounted for by the choice of a nonlinear eigenfunction expansion base. Also, the nonlinear equation of motion for the freezing front is solved together with the ordinary differential system for the integral transformed temperatures. After comparisons of the solution with previously reported numerical and experimental results, the influence of the related physical parameters on the droplet temperatures and freezing time is critically analysed.

## 1. Introduction

Problems involving supercooled droplets solidification find application in different fields, such as in the spray crystallization process [1,2], atmospheric research [3,4] and ice formation on surfaces [5–9]. Solidification is a rather complex process that involves the interplay of a number of physical effects [10]. The liquid is converted to ice through the crystallization process, which consists of two main steps, namely, nucleation and crystal growth [11]. The nucleation step comprises the formation of new crystals and can be homogeneous or heterogeneous. The crystal growth step occurs when the initial crystals from the nucleation step provide a structural pattern upon which all the material is deposited in the form of new crystals [11]. This last step is marked by the emergence of a solid/liquid interface. The solid/liquid interface is a moving boundary from which latent heat is released. This heat is conducted away from the interface through the material and then exchanged with the ambient.

Following the above concepts but aiming at more concise modelling, the freezing process of supercooled droplets is usually divided into four distinct stages: (i) supercooling stage, when the droplet cools from an initial temperature to the temperature at which the ice nucleation begins, known as nucleation temperature (*T_n_*); (ii) recalescence stage, which is kinetically controlled, involving partial freezing of the liquid and release of the latent heat, which then causes a quasi-instantaneous temperature rise in the whole droplet, taking its temperature from the nucleation temperature to the equilibrium freezing temperature (*T_f_*); (iii) freezing stage, when the ice crystals will grow until the droplet is fully solidified; and (iv) cooling stage, when the droplet cools from *T_f_*, towards the ambient air temperature if the droplet is suspended (or free), or to the substrate temperature if the droplet is lying on a substrate. The third stage of the process, the solidification stage, involves the solution of the convective–diffusive problem with a moving boundary due to the phase change, known in the literature as Stefan's problem. In the case of freezing of a spherical droplet immersed in a colder ambient, the moving boundary, also called the freezing front, moves from the outer surface of the droplet towards the centre, releasing latent heat. Stefan's problems can be formulated as one or two-phase problems. In the single-phase problem, the heat diffusion only occurs in one phase, while the other phase remains at a constant temperature equal to the equilibrium temperature for phase change. If conduction occurs within both phases, the problem is called a two-phase Stefan's problem.

Most of the pioneering works on this subject focused on using perturbation methods to obtain approximate solutions for one-dimensional Stefan's one-phase problem, considering the droplets initially at the freezing temperature [12–14]. Following these studies, Feuillebois *et al*. [15] analysed Stefan's one-phase problem by considering the freezing of liquid droplets immersed in cold air and subjected to supercooling. The perturbation method was used to obtain the evolution of the freezing front as a function of time, keeping the droplet surface temperature constant and equal to the external environment temperature. The authors postulated three different hypotheses for the location of the ice formed during nucleation: (a) at the centre of the droplet; (b) homogeneously distributed within the droplet; and (c) on the external surface of the droplet. The results obtained by hypotheses (a), (b) and (c) showed very similar results, and the result for hypothesis (b) was in between the results obtained by hypotheses (a) and (c). The solution obtained through the perturbation method was compared with the solution through a purely numerical approach, showing good agreement except in the region close to the centre of the droplet, where the perturbation method was not adherent to the numerical approach when hypotheses (b) and (c) were considered. Tabakova *et al*. [16] extended the work of Feuillebois *et al*. [15] for the case when the supercooled water droplets are subject to thermal convection on their surface. Again, a Stefan's one-phase problem was solved by the perturbation method, considering low Stefan numbers. The influence of convection on the freezing process was evaluated by varying the Biot number. As in Feuillebois *et al*. [15], the obtained perturbation solution deviated from a numerical solution based on the finite difference method under the enthalpy formulation when the droplet centre is approached. Outside this region, a good agreement with the numerical method is achieved. As expected, the results demonstrated a significant influence of the Biot number on the heat transfer within the droplet. The perturbation method was also applied to Stefan's two-phase problem for the solidification of spheres. McCue *et al*. [17] studied Stefan's two-phase problem for low Stefan numbers. The freezing time and temperature distribution in both phases were obtained considering the one-dimensional problem and a constant temperature condition on the outer surface of the sphere. An important conclusion of this study was that, under the conditions investigated, the influence of the liquid phase on the solidification process could be neglected. More recently, semi-analytical solutions have been used to solve Stefan's problem related to the ice crystal nucleation in the recalescence stage. Dragomirescu *et al*. [18] used a perturbation method as a starting solution, followed by a numerical method, to solve the one-dimensional Stefan's problem and, by combining these two methods, the authors were able to overcome the limitations of the perturbation method and to reduce the computational cost associated with using the numerical method alone. Schulte & Weigand [3] also employed a semi-analytical solution to study the same class of Stefan's problem, using a similarity solution based on a sub-scale model in order to deal with the different scales involved in the initial ice growth process in the recalescence stage.

Numerical methods were also extensively used to solve Stefan's problem related to droplet solidification [19–21]. Hindmarsh *et al*. [1] carried out a numerical–experimental study for the case of solidification of water droplets immersed in a cold air stream and suspended by a thermocouple. The freezing time was obtained for various values of ambient temperature, droplet size and airflow velocity. The abrupt change in temperature measured by the thermocouple and the change in droplet opacity, allowed the authors to experimentally determine the droplet nucleation temperature for each studied scenario. The values measured for the nucleation temperatures were then employed in the computer simulation. For the numerical study, it was assumed that the droplet keeps its spherical shape and therefore the equations can be analysed in a spherically symmetric formulation (i.e. with a single spatial dimension). Each of the four steps of the process mentioned above was analysed separately, and the solidification step was solved considering Stefan's two-phase problem. Two different models were considered in relation to the internal temperature distribution within the droplets, one considering the transient one-dimensional heat conduction, with moving boundary in the solidification stage, and the other considering the classical lumped system analysis (named uniform temperature method in [1]), in which the temperature of the droplets was assumed to be spatially uniform for the supercooling and cooling stages. For the solidification stage, the proposed approach involves a heat balance model, where the whole droplet freezes at a constant temperature (*T_f_*). The model considering heat conduction in the droplet was solved using the finite difference method, while the classical lumped model was directly solved by balancing the heat loss to the external environment with the latent and sensible heat released by the droplet. The solutions obtained for both models were compared with the experimental results, showing good agreement.

With the increasing interest in the application of hydrophobic coatings to mitigate the formation of ice on different structures, several studies have also been carried out on the freezing of water droplets deposited on surfaces [22–26]. Song *et al*. [22] made a comprehensive review of the experimental data associated with the characteristics of water droplets solidification on cold surfaces, providing an overview of important concepts involving the freezing process. The discussion of significant topics, such as freezing front shape, air bubbles formation and time scales of the process, is key for future research on this subject. Regarding numerical studies, many of them had the freezing of suspended water droplets as a starting point, since several conclusions obtained in those works extend to the case of freezing of water droplets on solid surfaces. Chaudhary & Li [23], for example, numerically studied the freezing of static droplets on surfaces subjected to rapid cooling and with different contact angles. As in the work of Hindmarsh *et al*. [1], the solidification process was divided into four distinct stages. Two-dimensional models were solved using the finite-element method and experiments were conducted for comparison with the numerical analysis, with the satisfactory agreement.

In the context of methodologies for solving moving boundary problems, hybrid numerical–analytical techniques have been gaining special attention in the literature. The generalized integral transform technique (GITT) is one of such hybrid techniques, and has been used successfully in solving various classes of diffusion and convection–diffusion problems [27–31], including the important class of problems with moving boundaries [32–34]. The GITT as applied to transient convection–diffusion involves the proposition of an eigenfunction expansion for the original potential spatial behaviour. Then, an analytical integral transformation is performed and an ordinary differential system in the time variable results for the unknown transformed potentials. In the most general situation, the nonlinear coupled structure of the ODE system requires a numerical treatment considering a truncated version of this infinite system, which characterizes the hybrid numerical–analytical nature of the methodology. The algorithm offers automatic error control together with a fairly modest overall computational effort, since the behaviour of the solution in all space variables is reconstructed analytically, while the main computational task is concentrated on the numerical solution of an ODE's initial value problem. The relative merits of both accuracy and computational cost were more closely discussed in the realm of classical test cases, such as in [35–37], but it should be noted that the advantages of the hybrid approach become even more evident as the number of spatial dimensions is increased, which affects mostly the analytical involvement, and when intensive computational tasks are considered, such as in optimization, inverse problem analysis and simulation under uncertainty, when a large number of evaluations of the direct problem is required. Some recent advances in the GITT have allowed for the use of techniques to further accelerate convergence in complex problems [38], and among these advances, we can mention the methodology variant introduced by Cotta *et al*. [39], where nonlinearities related to the boundary conditions of convection–diffusion problems are incorporated into the eigenvalue problem adopted in the integral transformation expansion base. This approach showed a significant convergence gain when compared with the more traditional GITT approach with a linear eigenvalue problem, in addition to presenting a uniform convergence behaviour over all the solution domain, even in positions close to the boundary conditions with nonlinear formulation [39].

The goal of this work is the extension and application of the GITT in simulating the solidification of supercooled water droplets immersed in a cold air stream. The droplets are considered to be freely suspended in air and maintaining their volume and spherical shape throughout the process, since the difference between ice and liquid droplet radii is less than about 3% when considering the typical values for liquid water and ice densities. The equations were written in spherical coordinates for one spatial dimension, by exploiting the spherical-symmetric configuration of the problem allowed for this simplification. The four stages of the process were sequentially analysed. The problem formulation takes into account the nonlinear effects of convection, radiation and convective mass transfer present on the surface of the droplets. In the solidification stage, Stefan's one-phase problem was solved, considering heat conduction in the solid phase only. For each of the stages, except the recalescence stage, GITT was applied to obtain the temperature variation with position and time, and to obtain the duration of each stage. The application of GITT in this class of problem provides more flexibility, in terms of solving complex nonlinear partial differential equations, compared with the approximate analytical methods previously used (e.g. perturbation methods), pulling out restrictions such as the range of the Stefan number that allows the achievement of a reliable solution, and at the same time offering a good computational precision-cost ratio, typically encountered in purely analytical methods. The nonlinear eigenvalue problem methodology introduced by Cotta *et al*. [39] was extended and combined with the use of implicit filters in order to enhance the solution's convergence rates, reducing furthermore the computational costs. This work brings some advances related to the GITT method itself, since the nonlinear eigenvalue methodology mentioned above was applied for the first time to moving boundary problems, together with the use of implicit filters, and taking into account highly nonlinear effects in the boundary conditions such as convective mass transfer and radiative heat transfer. The results obtained through the application of GITT were compared with numerical and experimental results previously reported in the literature. An analysis of the influence of the problem physical parameters on droplet temperatures and freezing times was also performed. The list and description of variables used in this work can be found in table 1.

**Table 1.** Nomenclature.

Symbol	Description
*a*(*τ*),*b*(*τ*)	coefficients for the boundary conditions
Bi*_c_*	characteristic Biot number for convective heat transfer
Bi*_m_*	characteristic Biot number for mass transfer
Bi*_r_*	dimensionless group for radiative heat transfer
*c*	specific heat (J kg^−1^ K^−1^)
*F*(*x,τ*)	filtering solutions
*h*	heat transfer coefficient (W m^−2^ K^−1^)
*h_m_*	mass transfer coefficient (m s^−1^)
*k*	thermal conductivity (W m^−1^ K^−1^)
*L*, *L_e_*, *L*_sb_	latent heats of solidification, evaporation and sublimation (J kg^−1^)
*L_x_*	latent heat of ice–water mixture (J kg^−1^)
*N*(*τ*)	normalization integral of the eigenvalue problem
*q*	heat flux (W m^−2^)
*R*	droplet radius (m)
*R_H_*	relative humidity
*R*_ini_	freezing front position after recalescence (m)
*r*	radial coordinate (m)
*s*(*t*)	freezing front position (m)
*St*	Stefan's number (= *c_s_*(*T_f_* − *T*_∞_)/*L*)
*t*	time variable (s)
*T*(*r,t*)	temperature distribution (K)
*T_f_*	freezing temperature (K)
*T_n_*	nucleation temperature (K)
*T_o_*	initial temperature (K)
*T_∞_*	ambient air temperature (K)
*U*_1_,*_o_*	ratio between initial temperature and ambient temperature
*U*	dimensionless temperature distribution
*v*	air flow velocity (m s^−1^)
*V*_dp_	droplet volume (m^3^)
*V_s_*	ice (solid phase) volume after recalescence (m^3^)
*x*, *y*	dimensionless space variables
Greek symbols	
*α*	thermal diffusivity (m^2^ s^−1^)
*ε*	emissivity
*δ_ij_*	Kronecker delta
*θ*(*x*,τ)	dimensionless temperature in Cartesian coordinates
*µ*	eigenvalues corresponding to eigenfunctions Ψ
*ν*(*τ*)	dimensionless freezing front position
*ρ*	density (kg m^−3^)
*ρ_v_*_,_*_o_*	water vapour density at 273 K (kg m^−3^)
*ρ_v_*_,_*_l_*, *ρ_v_*_,_*_s_*	water vapour density at liquid and solid droplet surfaces (kg m^−3^)
*ρ_v_*_,∞_	water vapour density at air surrounding the droplet (kg m^−3^)
*σ*	Stefan–Boltzmann constant
*τ*	dimensionless time variable
*ϕ*	liquid fraction in the water/ice mixture
*ψ*	eigenfunctions
subscripts and superscripts	
—	integral transformed variable
*	filtered potential
*i*, *j*	order of eigenquantities
*c*, *m*	related to convective heat or mass transfer, respectively
*l*, *s*	related to liquid and solid phases, respectively
*r*	related to radiation heat transfer
1 to 4	stages 1–4 (supercooling, recalescence, freezing, cooling)

## 2. Problem formulation

Due to space limitation, the full description of the dimensional problem formulation in all four stages is provided in the electronic supplementary material that accompanies the article, while here only the dimensionless forms are presented, without loss of content.

### (a) Supercooling (stage 1) and cooling stages (stage 4)

The supercooling and cooling stages are modelled using the one-dimensional heat conduction equation in spherical coordinates, considering constant thermophysical properties. The following dimensionless groups were chosen:

2.1 $$\begin{array}{rl} & U_{1}=\frac{T_{l}}{T_{\infty }}, \end{array}$$

2.2 $$\begin{array}{rl} & U_{1,o}=\frac{T_{o}}{T_{\infty }}, \end{array}$$

2.3 $$\begin{array}{rl} & x_{1}=\frac{r}{R}, \end{array}$$

2.4 $$\begin{array}{rl} & \alpha_{l}=\frac{k_{l}}{\left(c_{l}\rho_{l}\right)} \end{array}$$

2.5 $$\begin{array}{rl} \text{and}\; & \tau_{1}=\frac{\alpha_{l}t}{R^{2}}, \end{array}$$

where *T_l_* is the liquid (water) temperature, *k_l_* is the liquid thermal conductivity, *c_l_* is liquid specific heat, *ρ_l_* is the liquid specific mass, *r* is the radial distance from the droplet centre, *t* is time, *R* is the droplet external radius, *T*_∞_ is the air temperature and *T_o_* is the initial temperature. In addition, prior to the integral transform solution, the formulation can be rewritten similarly to the Cartesian coordinates system, by using the following classical variable transformation:

2.6 $$\theta_{1}=x_{1}U_{1}.$$

Then, the supercooling stage is written in dimensionless form as

2.7 $$\begin{array}{rl} & \frac{\partial^{2}\theta_{1}(x_{1},\tau_{1})}{\partial {x_{1}}^{2}}=\frac{\partial \theta_{1}(x_{1},\tau_{1})}{\partial \tau_{1}}\;\mathrm{for}\;0<x_{1}<1,\tau_{1}>0, \end{array}$$

2.8 $$\begin{array}{rl} & \theta_{1}(x_{1},0)=x_{1}U_{1,o}, \end{array}$$

2.9 $$\begin{array}{rl} & \theta_{1}(0,\tau_{1})=0, \end{array}$$

2.10 $$\begin{array}{rl} \mathrm{and}\; & {\frac{\partial \theta_{1}(x_{1},\tau_{1})}{\partial x_{1}}|}_{x_{1}=1}+B_{1}(\theta_{1}(1,\tau_{1}))\theta_{1}(1,\tau_{1})=H_{1}, \end{array}$$

with

2.11 $$\begin{array}{rl} & B_{1}(\theta_{1}(1,\tau_{1}))=Bi_{c,1}+Bi_{r,1}{\left(\theta_{1}(1,\tau_{1})\right)}^{3}+\frac{\rho_{v,l}(\theta_{1}(1,\tau_{1}))Bi_{m,1}}{\rho_{v,o}}-1, \end{array}$$

2.12 $$\begin{array}{rl} & H_{1}=Bi_{c,1}+Bi_{r,1}+\rho_{v,\infty }\frac{Bi_{m,1}}{\rho_{v,o}}, \end{array}$$

2.13 $$\begin{array}{rl} & \rho_{v,\infty }=R_{H}\frac{1.323}{T_{\infty }}e^{\left(19.83-5417/T_{\infty }\right)}, \end{array}$$

2.14 $$\begin{array}{rl} & \rho_{v,l}(\theta_{1}(1,\tau_{1}))=\frac{1.323}{T_{\infty }{\left(\theta_{1}(1,\tau_{1})\right)}^{2}}e^{\left(19.83-5417/T_{\infty }\theta_{1}(1,\tau_{1})\right)} \end{array}$$

2.15 $$\begin{array}{rl} & Bi_{c,1}=\frac{hR}{k_{l}}, \end{array}$$

2.16 $$\begin{array}{rl} & Bi_{m,1}=\frac{h_{m}L_{e}R\rho_{v,o}}{k_{l}T_{\infty }} \end{array}$$

2.17 $$\begin{array}{rl} \mathrm{and}\; & Bi_{r,1}=\frac{\varepsilon \sigma RT_{\infty }^{3}}{k_{l}}, \end{array}$$

where Bi*_c_*_,1_ represents the Biot number for convective heat transfer at stage 1, Bi*_m_*_,1_ is the Biot number for mass transfer, Bi*_r_*_,1_ is the dimensionless group related to the radiative heat transfer, *h* is the convection heat transfer coefficient, *h_m_* is the mass transfer coefficient, *L_e_* is the latent heat of evaporation, *ε* is the emissivity of the droplet surface, *σ* is the Stefan–Boltzmann constant, and *ρ_v_*_,*o*_ is the specific mass of the saturated water vapour at 273 K. For stage 1, when the droplet is in liquid state, *ρ_v_*_,∞_ and *ρ_v_*_,*l*_ are given by the relations above, equations (2.13–2.14) [40], where *R_H_* is the relative humidity of the air.

The cooling stage (stage 4) is modelled in a similar way as the supercooling stage above (stage 1). However, the thermophysical properties of the liquid phase need to be changed by the properties of the solid phase. Also, the initial temperature distribution, which for the supercooling stage (stage 1) is the uniform temperature *T_o_*, for the cooling stage (stage 4) it becomes the spatially varying temperature distribution in the droplet at the end of the solidification stage. Since the droplet is solid in this stage, the convective mass transfer occurs not through evaporation, but through sublimation, and therefore the latent heat of evaporation (*L_e_*) also needs to be substituted by the latent heat of sublimation (*L*_sb_). For stage 4 when the droplet is in solid state, *ρ_v_*_,*s*_ and *ρ_v_*_,∞_ are given by the following relations [40]:

2.18 $$\rho_{v,s}(T_{s}(R,t))=\frac{1.323}{T_{s}(R,t)}e^{\left(22.49-6141/T_{s}(R,t)\right)}$$

and

2.19 $$\rho_{v,\infty }=R_{H}\frac{1.323}{T_{\infty }}e^{\left(22.49-6141/T_{\infty }\right)}.$$

### (b) Recalescence stage (stage 2)

The recalescence stage is modelled as an adiabatic process, since the duration of this stage is considered to be very short, being much faster than the thermal diffusion through the droplet [22,23]. A global heat balance is adopted, following the relation proposed by Hindmarsh *et al*. [1], which is based on the assumption that the required heat to raise the droplet temperature from the nucleation temperature (*T_n_*) to the freezing temperature (*T_f_*), must be equal to the latent heat released to form the ice volume produced by nucleation. The relation proposed by Hindmarsh *et al*. [1] is given as

2.20 $$V_{s}=V_{\mathrm{dp}}c_{l}\rho_{l}\frac{\left(T_{f}-T_{n}\right)}{L\rho_{s}},$$

where *V_s_* is the solid (ice) volume formed, *V*_dp_ is the droplet volume, *c_l_* is the specific heat of the water in the liquid state, *T_f_* is the freezing temperature, *T_n_* is the nucleation temperature, *L* is the latent heat of solidification, *ρ_l_* is the liquid specific mass and *ρ_s_* is the solid specific mass.

In the present work, the ice volume formed after the recalescence stage (stage 2) will be modelled through two different paths. In the first case, the ice is formed as a spherical cask at the droplet surface and the initial position of the freezing front, *R*_ini_, is

2.21 $$R_{\mathrm{ini}}=\sqrt[{3}]{R^{3}-\frac{3V_{s}}{4\pi }}.$$

The second case considers a homogeneous distribution of the formed ice at the recalescence stage throughout the droplet. In this case, the water–ice mixture is considered as a uniform phase, and the initial interface position is at the droplet external surface, but the latent heat of solidification *L* is substituted by a new value for the water–ice mixture [16]. This latent heat of solidification and the liquid fraction in the mixture, ∅, are

2.22 $$L_{x}=\emptyset L$$

and

2.23 $$\emptyset =1-c_{l}\rho_{l}\frac{\left(T_{f}-T_{n}\right)}{\rho_{s}L}.$$

### (c) Freezing stage (stage 3)

The solidification stage is a heat conduction problem with a moving boundary, also known as Stefan's problem. The equations for the temperature distribution within the droplet and for the position of the freezing front must be solved simultaneously. The equation for the position of the freezing front, also known as Stefan's condition, describes the relationship between the speed of the interface and the removal of the latent heat released at the same interface [17] and will depend on the hypothesis adopted for the position of the ice formed in the recalescence stage, as will be shown in what follows. As mentioned earlier, this step will be solved as a one-phase Stefan problem and, therefore, the temperature of the liquid phase will be considered constant and equal to the freezing temperature *T_f_* and the conduction of the heat released in the freezing front will only occur in the solid phase. Similarly to the stage 1 formulation, this stage is modelled using the one-dimensional heat conduction equation in spherical coordinates, considering constant thermophysical properties. Once more, the original partial differential equations are recast in dimensionless form and the Cartesian coordinate system transformation is implemented. The following dimensionless groups were adopted:

2.24 $$\begin{array}{rl} & U_{3}=\frac{T_{s}-T_{f}}{T_{\infty }-T_{f}}, \end{array}$$

2.25 $$\begin{array}{rl} & y_{3}=\frac{r}{R}, \end{array}$$

2.26 $$\begin{array}{rl} & \alpha_{s}=\frac{k_{s}}{\left(c_{s}\rho_{s}\right)}, \end{array}$$

2.27 $$\begin{array}{rl} & v(\tau_{3})=\frac{s(\tau_{3})}{R}, \end{array}$$

2.28 $$\begin{array}{rl} & \tau_{3}=\frac{\alpha_{s}t}{R^{2}} \end{array}$$

2.29 $$\begin{array}{rl} \mathrm{and}\; & St=c_{s}\frac{\left(T_{f}-T_{\infty }\right)}{L}, \end{array}$$

where *T_s_* is the solid (ice) temperature, *k_s_* is the solid thermal conductivity, *c_s_* is the solid specific heat, *ρ_s_* is the solid specific mass, *r* is the radial distance from the droplet centre, *t* is time, *R* is the droplet external radius, *T*_∞_ is the air temperature, *s*(*τ*_3_) is the position of the freezing front, *L* is the latent heat of solidification and *St* is the Stefan number. The transformation to the Cartesian coordinates system is given by

2.30 $$\theta_{3}=y_{3}U_{3}$$

and it is also convenient to introduce an additional coordinate transformation in the dimensionless system as

2.31 $$x_{3}=1-y_{3}$$

and

2.32 $$\eta (\tau_{3})=1-v(\tau_{3}),$$

which leads to the following dimensionless problem formulation:

2.33 $$\begin{array}{rl} & \frac{\partial^{2}\theta_{3}(x_{3},\tau_{3})}{\partial {x_{3}}^{2}}=\frac{\partial \theta_{3}(x_{3},\tau_{3})}{\partial \tau_{3}}\;\mathrm{for}\;0<x_{3}<\eta (\tau_{3}),\tau_{3}>0, \end{array}$$

2.34 $$\begin{array}{rl} & \theta_{3}(x_{3},0)=0, \end{array}$$

2.35 $$\begin{array}{rl} & \theta_{3}(\eta (\tau_{3}),\tau_{3})=0, \end{array}$$

2.36 $$\begin{array}{rl} & -{\frac{\partial \theta_{3}(x_{3},\tau_{3})}{\partial x_{3}}|}_{x_{3}=0}+B_{3}(\theta_{3}(0,\tau_{3}))\theta_{3}(0,\tau_{3})=H_{3}, \end{array}$$

2.37a $$\begin{array}{rl} & {\frac{\partial \theta_{3}(x_{3},\tau_{3})}{\partial x_{3}}|}_{x_{3}=\eta (\tau_{3})}=-\frac{1}{St}(1-\eta (\tau_{3}))\frac{d\eta (\tau_{3})}{d\tau_{3}}, \end{array}$$

2.37b $$\begin{array}{rl} & \eta (0)=1-\frac{R_{\mathrm{ini}}}{R}, \end{array}$$

2.38a $$\begin{array}{r} {\frac{\partial \theta_{3}(x_{3},\tau_{3})}{\partial x_{3}}|}_{x_{3}=\eta (\tau_{3})}=-\frac{L_{x}}{L}\frac{1}{St}(1-\eta (\tau_{3}))\frac{d\eta (\tau_{3})}{d\tau_{3}}, \end{array}$$

2.38b $$\begin{array}{r} \eta (0)=0, \end{array}$$

2.39 $$\begin{array}{rl} & B_{3}(\theta_{3}(0,\tau_{3}))=Bi_{c,3}+4Bi_{r,3}\left(\left(1+\beta \theta_{3}(0,\tau_{3})+\frac{\beta^{2}{\left(\theta_{3}(0,\tau_{3})\right)}^{2}}{2}\right)\left(1+\frac{\beta \theta_{3}(0,\tau_{3})}{2}\right)\right)\\ & \;+\frac{\rho_{v,s}(\theta_{3}(0,\tau_{3}))Bi_{m,3}}{\rho_{v,o}}-1, \end{array}$$

2.40 $$\begin{array}{rl} & H_{3}=Bi_{c,3}-Bi_{r,3}\left(1-\frac{{T_{\infty }}^{4}}{{T_{f}}^{4}}\right)\frac{1}{\beta }+\rho_{v,\infty }\frac{Bi_{m,3}}{\rho_{v,o}}, \end{array}$$

2.41 $$\begin{array}{rl} & \rho_{v,s}(\theta_{3}(0,\tau_{3}))=\frac{1.323}{\left((T_{\infty }-T_{f})\theta_{3}(0,\tau_{3})+T_{f})\theta_{3}(0,\tau_{3}\right)}e^{\left(22.49-6141/(T_{\infty }-T_{f})\theta_{3}(0,\tau_{3})+T_{f}\right)} \end{array}$$

2.42 $$\begin{array}{rl} & Bi_{c,3}=\frac{hR}{k_{s}}, \end{array}$$

2.43 $$\begin{array}{rl} & Bi_{m,3}=\frac{h_{m}L_{sb}R\rho_{v,o}}{k_{s}(T_{\infty }-T_{f})}, \end{array}$$

2.44 $$\begin{array}{rl} & Bi_{r,3}=\frac{\varepsilon \sigma RT_{f}^{3}}{k_{s}} \end{array}$$

2.45 $$\begin{array}{rl} \mathrm{and}\; & \beta =\frac{T_{\infty }-T_{f}}{T_{f}}, \end{array}$$

where Bi*_c_*_,3_ is the Biot number for convective heat transfer in stage 3, Bi*_m_*_,3_ is the Biot number for mass transfer, Bi*_r_*_,3_ is the dimensionless group for radiative heat transfer, *h* is the convective heat transfer coefficient, *h_m_* is the mass transfer coefficient, *L*_sb_ is the latent heat of sublimation, *ε* is the emissivity, *σ* is the Stefan–Boltzmann constant and *ρ_v_*_,*o*_ is the specific mass of saturated water vapour at 273 K. Equations (2.37*a*) and (2.37*b*) represent the formulation for the moving boundary related to the hypothesis of the formation of a spherical cask at the droplet surface, while equations (2.38*a*) and (2.38*b*) represent the formulation related to the hypothesis of a uniform distribution of the initially formed ice.

## 3. Solution methodology

The above dimensionless problem formulations are now solved by the GITT for each stage governed by partial differential equations (except the recalescence stage). For convergence enhancement, the nonlinearities in boundary conditions shall be directly incorporated into the chosen eigenvalue problem, as originally proposed by Cotta *et al*. [39]. Implicit filters are also adopted in order to homogenize the nonlinear boundary conditions at the external surface of the droplet.

### (a) Supercooling stage (stage 1)

The solution of the supercooling stage is started with filtering the dimensionless form of the problem formulation. With the objective of homogenizing the boundary condition of equation (2.10), the following implicit filtering solution is proposed:

3.1 $$\theta_{1}(x_{1},\tau_{1})=F_{1}(x_{1},\tau_{1})+\theta_{1}^{*}(x_{1},\tau_{1}),$$

where *F*_1_ is the proposed filter and $\theta_{1}^{*}$ is the dimensionless filtered potential. A plain linear function in *x*_1_ has been adopted as filter, given as

3.2 $$F_{1}(x_{1},\tau_{1})=a_{1}(\tau_{1})x_{1}+b_{1}(\tau_{1}),$$

where *a*_1_(*τ*_1_) and *b*_1_(*τ*_1_) are coefficients that ensure the satisfaction of the nonlinear boundary conditions, while the time variable *τ*_1_ functions as a parameter in the filter equation. After applying the filter (equation (3.1)) to the dimensionless problem (equations (2.7)–(2.10)), the following filtered problem is achieved:

3.3 $$\begin{array}{rl} & \frac{\partial^{2}\theta_{1}^{*}(x_{1},\tau_{1})}{\partial {x_{1}}^{2}}=\frac{\partial \theta_{1}^{*}(x_{1},\tau_{1})}{\partial \tau_{1}}+x_{1}\frac{da_{1}(\tau_{1})}{d\tau_{1}}\;\mathrm{for}\;0<x_{1}<1,\tau_{1}>0, \end{array}$$

3.4 $$\begin{array}{rl} & \theta_{1}^{*}(x_{1},0)=x_{1}(U_{1,o}-a_{1}(0)), \end{array}$$

3.5 $$\begin{array}{rl} & \theta_{1}^{*}(0,\tau_{1})=0, \end{array}$$

3.6 $$\begin{array}{rl} & {\frac{\partial \theta_{1}^{*}(x_{1},\tau_{1})}{\partial x_{1}}|}_{x_{1}=1}+B_{1}(a_{1}(\tau_{1})+\theta_{1}^{*}(1,\tau_{1}))\theta_{1}^{*}(1,\tau_{1})=0, \end{array}$$

3.7 $$\begin{array}{rl} & B_{1}(a_{1}(\tau_{1})+\theta_{1}^{*}(1,\tau_{1}))=Bi_{c,1}+Bi_{r,1}{\left(a_{1}(\tau_{1})+\theta_{1}^{*}(1,\tau_{1})\right)}^{3}\\ & \;\;\;\;\;\;+\frac{\rho_{v,l}(a_{1}(\tau_{1})+\theta_{1}^{*}(1,\tau_{1}))Bi_{m,1}}{\rho_{v,o}}-1, \end{array}$$

3.8 $$\begin{array}{rl} & \rho_{v,l}(a_{1}(\tau_{1})+\theta_{1}^{*}(1,\tau_{1}))=\frac{1.323}{T_{\infty }{\left(a_{1}(\tau_{1})+\theta_{1}^{*}(1,\tau_{1})\right)}^{2}}e^{\left(19.83-5417/T_{\infty }(a_{1}(\tau_{1})+\theta_{1}^{*}(1,\tau_{1}))\right)}, \end{array}$$

3.9 $$\begin{array}{rl} & a_{1}(\tau_{1})=\frac{H_{1}}{1+B_{1}(a_{1}(\tau_{1})+\theta_{1}^{*}(1,\tau_{1}))} \end{array}$$

3.10 $$\begin{array}{rl} \mathrm{and}\; & b_{1}(\tau_{1})=0. \end{array}$$

Then, the GITT approach is employed in the solution of the above-filtered problem, starting with the choice of the eigenvalue problem and the corresponding transform-inverse pair. The eigenvalue problem is proposed from inspection of the homogeneous filtered problem, equations ((3.3)–(3.6)). Following the methodology introduced in Cotta *et al*. [39] for convergence enhancement, the proposed nonlinear eigenvalue problem incorporates the nonlinear coefficient in the boundary condition, equation (3.6). Differently from the traditional approach, the eigenvalues and eigenfunctions become functions of the original potential and thus of the time variable. The integral transform pair and the eigenvalue problem are then given by

3.11 $$\begin{array}{rl} & {\bar{\theta_{1}^{*}}}_{i}(\tau_{1})=\int_{0}^{1}{\psi_{1}}_{i}(x_{1},\tau_{1})\theta_{1}^{*}(x_{1},\tau_{1})dx_{1}\;\to \;\mathrm{Transform}, \end{array}$$

3.12 $$\begin{array}{rl} & \theta_{1}^{*}(x_{1},\tau_{1})=\sum_{i=1}^{\infty }\frac{{\bar{\theta_{1}^{*}}}_{i}(\tau_{1}){\psi_{1}}_{i}(x_{1},\tau_{1})}{{N_{1}}_{i}(\tau_{1})}\;\to \;\mathrm{Inverse}, \end{array}$$

3.13 $$\begin{array}{rl} & \frac{\partial^{2}\psi_{1_{i}}(x_{1},\tau_{1})}{\partial {x_{1}}^{2}}+{\left({\mu_{1}}_{i}(\tau_{1})\right)}^{2}{\psi_{1}}_{i}(x_{1},\tau_{1})=0\;\mathrm{for}\;0<x_{1}<1, \end{array}$$

3.14 $$\begin{array}{rl} & \psi_{1}(0,\tau_{1})=0 \end{array}$$

3.15 $$\begin{array}{rl} \mathrm{and}\; & {\frac{\partial {\psi_{1}}_{i}(x_{1},\tau_{1})}{\partial x_{1}}|}_{x_{1}=1}+B_{1}(a_{1}(\tau_{1})+\theta_{1}^{*}(1,\tau_{1})){\psi_{1}}_{i}(1,\tau_{1})=0, \end{array}$$

where *μ*_1*i*_(*τ*_1_) represents the eigenvalues and *ψ*_1*i*_(*x*_1_, *τ*_1_) are the eigenfunctions, with analytical solutions for eigenfunctions, norms and transcendental equation given by

3.16 $$\begin{array}{rl} & {\psi_{1}}_{i}(x_{1},\tau_{1})=\sin({\mu_{1}}_{i}(\tau_{1})x_{1}), \end{array}$$

3.17 $$\begin{array}{rl} & {N_{1}}_{i}(\tau_{1})=\int_{0}^{1}{\left({\psi_{1}}_{i}(x_{1},\tau_{1})\right)}^{2}dx_{1} \end{array}$$

3.18 $$\begin{array}{rl} \mathrm{and}\; & {\mu_{1}}_{i}(\tau_{1})\cos({\mu_{1}}_{i}(\tau_{1}))+B_{1}(a_{1}(\tau_{1})+\theta_{1}^{*}(1,\tau_{1}))\sin({\mu_{1}}_{i}(\tau_{1}))=0. \end{array}$$

Proceeding to the integral transformation process, the operator $\int_{0}^{1}{\psi_{1}}_{i}\_\_\_\_\_\_\_dx_{1}$ is applied to equation (3.3), and after some manipulations involving the boundary conditions, the following transformed ordinary differential system is obtained:

3.19 $$\begin{array}{rl} & \frac{d{\bar{\theta_{1}^{*}}}_{i}(\tau_{1})}{d\tau_{1}}+\sum_{j=1}^{\infty }\left(\delta_{ij}{\left({\mu_{1}}_{i}(\tau_{1})\right)}^{2}-\int_{0}^{1}\frac{{\psi_{1}}_{j}(x_{1},\tau_{1})}{{N_{1}}_{j}(\tau_{1})}\frac{\partial {\psi_{1}}_{i}(x_{1},\tau_{1})}{\partial \tau_{1}}dx_{1}\right){\bar{\theta_{1}^{*}}}_{j}(\tau_{1})\\ & \;=-\frac{da_{1}(\tau_{1})}{d\tau_{1}}\int_{0}^{1}{\psi_{1}}_{i}(x_{1},\tau_{1})x_{1}dx_{1} \end{array}$$

and

3.20 $${\bar{\theta_{1}^{*}}}_{i}(0)=(U_{1,o}-a_{1}(0))\int_{0}^{1}{\psi_{1}}_{i}(x_{1},0)x_{1}dx_{1},$$

where the filter coefficient *a*_1_(*τ*_1_) should be simultaneously solved with the transformed system. One possible path for obtaining the eigenvalues *μ*_1*i*_(*τ*_1_) is to differentiate equation (3.18) with respect to *τ*_1_, and employ equation (3.18) for *τ*_1 _= 0 as an initial condition. Thus, the ODE system for the eigenvalues is written as

3.21 $$\begin{array}{rl} & \frac{d{\mu_{1}}_{i}(\tau_{1})}{d\tau_{1}}(\cos({\mu_{1}}_{i}(\tau_{1}))-{\mu_{1}}_{i}(\tau_{1})\sin({\mu_{1}}_{i}(\tau_{1}))+B_{1}(a_{1}(\tau_{1})+\theta_{1}^{*}(1,\tau_{1}))\cos({\mu_{1}}_{i}(\tau_{1})))\\ & \;+\frac{dB_{1}(a_{1}(\tau_{1})+\theta_{1}^{*}(1,\tau_{1}))}{d\tau_{1}}\sin({\mu_{1}}_{i}(\tau_{1}))=0 \end{array}$$

and

3.22 $${\mu_{1}}_{i}(0)\cos({\mu_{1}}_{i}(0))+B_{1}(a_{1}(0)+\theta_{1}^{*}(1,0))\sin({\mu_{1}}_{i}(0))=0.$$

Similarly, the differential equation for the coefficient *a*_1_(*τ*_1_) is obtained by differentiating equation (3.9) with respect to *τ*_1_, and employing equation (3.9) at *τ*_1_ = 0 as an initial condition, yielding:

3.23 $$\frac{da_{1}(\tau_{1})}{d\tau_{1}}(1+B_{1}(a_{1}(\tau_{1})+\theta_{1}^{*}(1,\tau_{1})))+\frac{dB_{1}(a_{1}(\tau_{1})+\theta_{1}^{*}(1,\tau_{1}))}{d\tau_{1}}a_{1}(\tau_{1})=0$$

and

3.24 $$a_{1}(0)=\frac{H_{1}}{1+B_{1}(a_{1}(0)+\theta_{1}^{*}(1,0))}.$$

Equations ((3.19)–(3.22)) and ((3.23)–(3.24)) form a coupled system of nonlinear ODEs to be numerically solved upon truncation to a finite order *M*, sufficiently large to warrant convergence in the desired potentials. The filtered temperature, $\theta_{1}^{*}(x_{1},\tau_{1})$, is then analytically obtained from the inverse formula (equation (3.12)) from knowledge of the transformed temperatures ${\bar{\theta_{1}^{*}}}_{i}(\tau_{1})$.

### (b) Recalescence stage (stage 2)

As previously discussed, the recalescence stage is assumed to occur instantaneously, and the only computation required is the total ice volume formed by nucleation, once this quantity affects the values of *ϕ* and *R*_ini_. As can be seen from equation (2.20), this quantity is algebraically determined, and does not require any specific mathematical method.

### (c) Freezing stage (stage 3)

Again, an implicit filter is proposed to homogenize the nonlinear boundary condition, equation (2.36), as

3.25 $$\theta_{3}(x_{3},\tau_{3})=F_{3}(x_{3},\tau_{3})+\theta_{3}^{*}(x_{3},\tau_{3})$$

and

3.26 $$F_{3}(x_{3},\tau_{3})=a_{3}(\tau_{3})x_{3}+b_{3}(\tau_{3}),$$

where *F*_3_ is the proposed filter with a linear functional behaviour in *x*_3_ and $\theta_{3}^{*}$ is the filtered potential, *a*_3_(*τ*_3_) and *b*_3_(*τ*_3_) are coefficients to be determined that warrant elimination of the boundary condition source term. The filter is then applied to equations ((2.33)–(2.36)), yielding:

3.27 $$\begin{array}{rl} & \frac{\partial^{2}\theta_{3}^{*}(x_{3},\tau_{3})}{\partial {x_{3}}^{2}}=\frac{\partial \theta_{3}^{*}(x_{3},\tau_{3})}{\partial \tau_{3}}+x_{3}\frac{da_{3}(\tau_{3})}{d\tau_{3}}+\frac{db_{3}(\tau_{3})}{d\tau_{3}},0<x_{3}<\eta (\tau_{3}),\tau_{3}>0, \end{array}$$

3.28 $$\begin{array}{rl} & \theta_{3}^{*}(x_{3},0)=-a_{3}(0)x_{3}-b_{3}(0), \end{array}$$

3.29 $$\begin{array}{rl} & -{\frac{\partial \theta_{3}^{*}(x_{3},\tau_{3})}{\partial x_{3}}|}_{x_{3}=0}+B_{3}(b_{3}(\tau_{3})+\theta_{3}^{*}(0,\tau_{3}))\theta_{3}^{*}(0,\tau_{3})=0, \end{array}$$

3.30 $$\begin{array}{rl} & \theta_{3}^{*}(\eta (\tau_{3}),\tau_{3})=0 \end{array}$$

3.31 $$\begin{array}{rl} & B_{3}(b_{3}(\tau_{3})+\theta_{3}^{*}(0,\tau_{3}))=Bi_{c,3}+4Bi_{r,3}(\left(1+\beta (b_{3}(\tau_{3})+\theta_{3}^{*}(0,\tau_{3}))+\frac{\beta^{2}{\left(b_{3}(\tau_{3})+\theta_{3}^{*}(0,\tau_{3})\right)}^{2}}{2}\right)\\ & \;+\left(1+\frac{\beta (b_{3}(\tau_{3})+\theta_{3}^{*}(0,\tau_{3}))}{2}\right))+\frac{\rho_{v,s}(b_{3}(\tau_{3})+\theta_{3}^{*}(0,\tau_{3}))Bi_{m,3}}{\rho_{v,o}}-1, \end{array}$$

3.32 $$\begin{array}{rl} & a_{3}(\tau_{3})=\frac{-H_{3}}{1+B_{3}(b_{3}(\tau_{3})+\theta_{3}^{*}(0,\tau_{3}))\eta (\tau_{3})}, \end{array}$$

3.33 $$\begin{array}{rl} & b_{3}(\tau_{3})=\frac{H_{3}\eta (\tau_{3})}{1+B_{3}(b_{3}(\tau_{3})+\theta_{3}^{*}(0,\tau_{3}))\eta (\tau_{3})} \end{array}$$

3.34 $$\begin{array}{rl} \mathrm{and}\; & \rho_{v,s}(b_{3}(\tau_{3})+\theta_{3}^{*}(0,\tau_{3})))\\ & \;=\left(\frac{1.323}{\begin{array}{l} \left((T_{\infty }-T_{f})(b_{3}(\tau_{3})+\theta_{3}^{*}(0,\tau_{3}))+T_{f}\right)\\ \;\times (b_{3}(\tau_{3})+\theta_{3}^{*}(0,\tau_{3})) \end{array}}\right)e^{\left(22.49-6141/((T_{\infty }-T_{f})(b_{3}(\tau_{3})+\theta_{3}^{*}(0,\tau_{3}))+T_{f})\right)}. \end{array}$$

The filtered problem is again solved using GITT, once more adopting the nonlinear eigenfunction expansion base. Thus, the integral transform pair and the eigenvalue problem, now with a moving boundary, were chosen as

3.35 $$\begin{array}{rl} & {\bar{\theta_{3}^{*}}}_{i}(\tau_{3})=\int_{0}^{\eta (\tau_{3})}{\psi_{3}}_{i}(x_{3},\tau_{3})\theta_{3}^{*}(x_{3},\tau_{3})dx_{3}\;\to \;\mathrm{Transform}. \end{array}$$

3.36 $$\begin{array}{rl} & \theta_{3}^{*}(x_{3},\tau_{3})=\sum_{i=1}^{\infty }\frac{{\bar{\theta_{3}^{*}}}_{i}(\tau_{3}){\psi_{3}}_{i}(x_{3},\tau_{3})}{{N_{3}}_{i}(\tau_{3})}\;\to \;\mathrm{Inverse}, \end{array}$$

3.37 $$\begin{array}{rl} & \frac{\partial^{2}{\psi_{3}}_{i}(x_{3},\tau_{3})}{\partial {x_{3}}^{2}}+{\left({\mu_{3}}_{i}(\tau_{3})\right)}^{2}{\psi_{3}}_{i}(x_{3},\tau_{3})=0\;\mathrm{for}\;0<x_{3}<\eta (\tau_{3}), \end{array}$$

3.38 $$\begin{array}{rl} & -{\frac{\partial {\psi_{3}}_{i}(x_{3},\tau_{3})}{\partial x_{3}}|}_{x_{3}=0}+B_{3}(b_{3}(\tau_{3})+\theta_{3}^{*}(0,\tau_{3})){\psi_{3}}_{i}(0,\tau_{3})=0 \end{array}$$

3.39 $$\begin{array}{rl} \mathrm{and}\; & {\psi_{3}}_{i}(\eta (\tau_{3}),\tau_{3})=0. \end{array}$$

The eigenvalue problem is analytically solved to yield:

3.40 $$\begin{array}{rl} & {\psi_{3}}_{i}(x_{3},\tau_{3})=\sin({\mu_{3}}_{i}(\tau_{3})(\eta (\tau_{3})-x_{3})), \end{array}$$

3.41 $$\begin{array}{rl} & {N_{3}}_{i}(\tau_{3})=\int_{0}^{\eta (\tau_{3})}{\left({\psi_{3}}_{i}(x_{3},\tau_{3})\right)}^{2}dx_{3} \end{array}$$

3.42 $$\begin{array}{rl} \mathrm{and}\; & {\mu_{3}}_{i}(\tau_{3})\cos({\mu_{3}}_{i}(\tau_{3})\eta (\tau_{3}))+B_{3}(b_{3}(\tau_{3})+\theta_{3}^{*}(0,\tau_{3}))\sin({\mu_{3}}_{i}(\tau_{3})\eta (\tau_{3}))=0. \end{array}$$

The integral operator $\int_{0}^{\eta (\tau_{3})}{\psi_{3}}_{i}\_\_\_\_dx_{3}$ is applied on both sides of equation (3.27), and after some manipulations, the transformed ODE system is obtained as

3.43 $$\begin{array}{rl} & \frac{d{\bar{\theta_{3}^{*}}}_{i}(\tau_{3})}{d\tau_{3}}+\sum_{j=1}^{\infty }\left(\delta_{ij}{\left({\mu_{3}}_{i}(\tau_{3})\right)}^{2}-\int_{0}^{\eta (\tau_{3})}\frac{{\psi_{3}}_{j}(x_{3},\tau_{3})}{{N_{3}}_{j}(\tau_{3})}\frac{\partial {\psi_{3}}_{i}(x_{3},\tau_{3})}{\partial \tau_{3}}dx_{3}\right){\bar{\theta_{3}^{*}}}_{j}(\tau_{3})\\ & \;=-\frac{da_{3}(\tau_{3})}{d\tau_{3}}\int_{0}^{\eta (\tau_{3})}{\psi_{3}}_{i}(x_{3},\tau_{3})x_{3}dx_{3}-\frac{db_{3}(\tau_{3})}{d\tau_{3}}\int_{0}^{\eta (\tau_{3})}{\psi_{3}}_{i}(x_{3},\tau_{3})dx_{3} \end{array}$$

and

3.44 $${\bar{\theta_{3}^{*}}}_{i}(0)=-a_{3}(0)\int_{0}^{\eta (\tau_{3})}{\psi_{3}}_{i}(x_{3},\tau_{3})x_{3}dx_{3}-b_{3}(0)\int_{0}^{\eta (\tau_{3})}{\psi_{3}}_{i}(x_{3},\tau_{3})dx_{3}.$$

The coefficients *a*_3_(*τ*_3_) and *b*_3_(*τ*_3_) shall be solved simultaneously with the transformed potentials.

The moving boundary equation is now considered, by substituting the filter expression, equations ((3.25)–(3.26)), and the inverse formula, equation (3.36), into equations (2.37) and (2.38), yielding:

3.45a $$\begin{array}{rl} & \frac{\partial }{\partial x_{3}}{\left(\overset{\infty }{\underset{j=1}{\sum }}\frac{{\bar{\theta_{3}^{*}}}_{j}(\tau_{3}){\psi_{3}}_{j}(x_{3},\tau_{3})}{{N_{3}}_{j}(\tau_{3})}\right)|}_{x_{3}=\eta (\tau_{3})}+a_{3}(\tau_{3})=-\frac{1}{St}(1-\eta (\tau_{3}))\frac{d\eta (\tau_{3})}{d\tau_{3}}, \end{array}$$

3.45b $$\begin{array}{rl} & \eta (0)=1-\frac{R_{\mathrm{ini}}}{R}, \end{array}$$

3.46a $$\begin{array}{rl} & \frac{\partial }{\partial x_{3}}{\left(\overset{\infty }{\underset{j=1}{\sum }}\frac{{\bar{\theta_{3}^{*}}}_{j}(\tau_{3}){\psi_{3}}_{j}(x_{3},\tau_{3})}{{N_{3}}_{j}(\tau_{3})}\right)|}_{x_{3}=\eta (\tau_{3})}+a_{3}(\tau_{3})=-\frac{L_{x}}{L}\frac{1}{St}(1-\eta (\tau_{3}))\frac{d\eta (\tau_{3})}{d\tau_{3}} \end{array}$$

3.46b $$\begin{array}{rl} \mathrm{and}\; & \eta (0)=0. \end{array}$$

As for the supercooling stage, an ODE system is assembled to solve for the eigenvalues, *μ*_3*i*_(*τ*_3_), by differentiating the corresponding transcendental equation, equation (3.42), with respect to *τ*_3_. The corresponding ODEs and initial conditions at *τ*_3 _= 0, are given by

3.47 $$\begin{array}{rl} & \frac{d{\mu_{3}}_{i}(\tau_{3})}{d\tau_{3}}(\cos({\mu_{3}}_{i}(\tau_{3})\eta (\tau_{3}))-{\mu_{3}}_{i}(\tau_{3})\eta (\tau_{3})\sin({\mu_{3}}_{i}(\tau_{3})\eta (\tau_{3}))\\ & \;+B_{3}(b_{3}(\tau_{3})+\theta_{3}^{*}(0,\tau_{3}))\eta (\tau_{3})\cos({\mu_{3}}_{i}(\tau_{3})\eta (\tau_{3})))\\ & \;-{\left({\mu_{3}}_{i}(\tau_{3})\right)}^{2}\sin({\mu_{3}}_{i}(\tau_{3})\eta (\tau_{3}))\frac{d\eta (\tau_{3})}{d\tau_{3}}\\ & \;+B_{3}(b_{3}(\tau_{3})+\theta_{3}^{*}(0,\tau_{3})){\mu_{3}}_{i}(\tau_{3})\cos({\mu_{3}}_{i}(\tau_{3})\eta (\tau_{3}))\frac{d\eta (\tau_{3})}{d\tau_{3}}\\ & \;+\frac{dB_{3}(b_{3}(\tau_{3})+\theta_{3}^{*}(0,\tau_{3}))}{d\tau_{3}}\sin({\mu_{3}}_{i}(\tau_{3})\eta (\tau_{3}))=0 \end{array}$$

and

3.48 $${\mu_{3}}_{i}(0)\cos({\mu_{3}}_{i}(0)\eta (0))+B_{3}(b_{3}(0)+\theta_{3}^{*}(0,0))\sin({\mu_{3}}_{i}(0)\eta (0))=0.$$

Similarly, the ODEs for obtaining the coefficients *a*_3_(*τ*_3_) and *b*_3_(*τ*_3_) are obtained with the respective initial conditions:

3.49 $$\begin{array}{rl} & \frac{da_{3}(\tau_{3})}{d\tau_{3}}(1+B_{3}(b_{3}(\tau_{3})+\theta_{3}^{*}(0,\tau_{3}))\eta (\tau_{3}))+\frac{dB_{3}(b_{3}(\tau_{3})+\theta_{3}^{*}(0,\tau_{3}))}{d\tau_{3}}a_{3}(\tau_{3})\eta (\tau_{3})\\ & \;+a_{3}(\tau_{3})B_{3}(b_{3}(\tau_{3})+\theta_{3}^{*}(0,\tau_{3}))\frac{d\eta (\tau_{3})}{d\tau_{3}}=0, \end{array}$$

3.50 $$\begin{array}{rl} & a_{3}(0)=\frac{-H_{3}}{1+B_{3}(b_{3}(0)+\theta_{3}^{*}(0,0))\eta (0)}, \end{array}$$

3.51 $$\begin{array}{rl} & \frac{db_{3}(\tau_{3})}{d\tau_{3}}(1+B_{3}(b_{3}(\tau_{3})+\theta_{3}^{*}(0,\tau_{3}))\eta (\tau_{3}))\\ & \;+\frac{dB_{3}(b_{3}(\tau_{3})+\theta_{3}^{*}(0,\tau_{3}))}{d\tau_{3}}b_{3}(\tau_{3})\eta (\tau_{3})\\ & \;+b_{3}(\tau_{3})B_{3}(b_{3}(\tau_{3})+\theta_{3}^{*}(0,\tau_{3}))\frac{d\eta (\tau_{3})}{d\tau_{3}}-H_{3}\frac{d\eta (\tau_{3})}{d\tau_{3}}=0 \end{array}$$

3.52 $$\begin{array}{rl} \mathrm{and}\; & b_{3}(0)=\frac{H_{3}\eta (0)}{1+B_{3}(b_{3}(0)+\theta_{3}^{*}(0,0))\eta (0)}. \end{array}$$

The coupled system of ODEs in equations ((3.43)–(3.44)), ((3.47)–(3.48)) and ((3.49)–(3.52)) is then numerically solved for a truncated version of the system with a finite order *M*. Depending on the adopted hypothesis for ice formation structure in the recalescence stage, either equation (3.45) or (3.46) should be added to the system. Then, the dimensionless temperature distribution within the droplet, $\theta_{3}^{*}(x_{3},\tau_{3})$, is obtained from the inverse formula, equation (3.36), after the transformed potentials, ${\bar{\theta_{3}^{*}}}_{i}(\tau_{3})$ have been numerically computed. The solidification interface position, *η*(*τ*_3_), is directly obtained from the numerical solution of the ODE system, and the dimensional boundary position is finally determined from:

3.53 $$s(\tau_{3})=R(1-\eta (\tau_{3})).$$

### (d) Cooling stage (stage 4)

The solution methodology for this stage is the same as for the supercooling stage (stage 1), with the necessary changes in the initial condition and other parameters, as discussed in the problem formulation section, and skipped here for brevity.

## 4. Results and discussion

This section provides validation and verification of the proposed model and integral transform solution, followed by some parametric analysis. First, a simpler model proposed by Tabakova *et al*. [16], which considers only convection at the external surface of the droplet and has been solved by both approximate analytical and numerical methods, will be considered and compared with results obtained from the present analysis. Only the solidification stage is then verified. Second, the present complete model shall be compared with the experimental and numerical results of Hindmarsh *et al*. [1], considering all the relevant effects at the droplet surface, namely, convection, radiation and convective mass transfer. Finally, a physical analysis of the most relevant parameters on the overall process is undertaken. The routine *NDSolve* from the *Mathematica* v. 11.3 system has been employed in the numerical solution of the transformed ODEs system with automatic accuracy and precision control.

### (a) Comparison with perturbation and numerical methods

The more general model presented above is first simplified by neglecting the nonlinear terms related to radiative heat transfer and convective mass transfer. The results for the solidification stage are then compared with both numerical (finite difference method with enthalpy formulation) and perturbation methods extracted from the work of Tabakova *et al*. [16], for different values of the Stefan number (*St*) and the Biot number (Bi*_c_*_,3_). In the GITT simulation, convergence rates were first analysed in terms of the dimensionless temperature distribution, *U*_3_(*y*_3_, *τ*_3_), and the dimensionless freezing time, and full convergence to at least four significant digits was achieved for truncation orders as low as *M* = 20, as illustrated in table 2 below for Bi*_c_*_,3 _= 1 and *St* = 0.1, and different truncation orders *M*.

**Table 2.** The freezing time and dimensionless temperature at the droplet surface, for Bi*_c_*_,3_ = 1 and *St *= 0.1, resulting from the GITT solution for different truncation orders (*M*). Convergence to at least four digits is achieved for *M* as low as 20.

*M*	*τ*_3_	*U*_3_ [1, 0.5]	*U*_3_ [1, 0.1]
10	0.5337	1.2979	1.8945
15	0.5341	1.2983	1.8945
20	0.5342	1.2984	1.8945
25	0.5342	1.2984	1.8945

**Figure 1.**
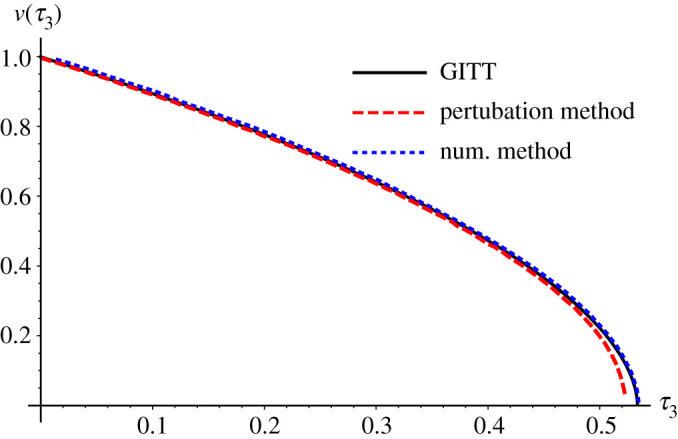
Comparisons of dimensionless interface position *v*(*τ*_3_): GITT hybrid solution (*M *= 20) (black solid line), finite difference method with enthalpy formulation [16] (blue dotted line) and perturbation method [16] (dashed red line) for Bi*_c_*_,3 _= 1 and *St* = 0.1. (Online version in colour.)

**Figure 2.**
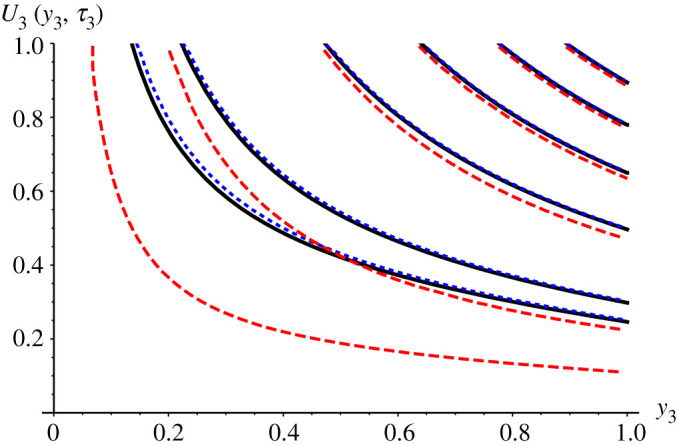
Comparisons of dimensionless temperature *U*_3_(*y*_3_, *τ*_3_): GITT hybrid solution (*M* = 20) (black solid lines), finite difference method with enthalpy formulation [16] (blue dotted line) and perturbation method [16] (red dashed lines) at *τ*_3 _= 0.1, 0.2, 0.3, 0.4, 0.5 and 0.52 from right to left, for Bi*_c_*_,3 _= 1 and *St* = 0.1. (Online version in colour.)

Figures 1 and 2 provide a comparison of the results from Tabakova *et al*. [16] for *St* = 0.1 and Bi*_c_*_,3 _= 1 with the GITT solution for a fixed truncation order of *M* = 20. Figure 1 shows results for the dimensionless freezing front position, *v*(*τ*_3_), with excellent agreement between the GITT and the numerical method along the entire time domain. The perturbation method solution, however, presents some deviation by the end of the freezing process. Figure 2 compares the dimensionless temperature profiles in the solid phase for different values of *τ*_3_. Again it can be observed that the GITT solution agrees notably well with the numerical solution based on the finite difference method with enthalpy formulation. Once more the perturbation method presents marked discrepancies as the end of the solidification stage is approached. In fact, according to Tabakova *et al*. [16], for a large Stefan number and close to the freezing time, the perturbation solution becomes less consistent, and the values *τ*_3 _= 0.5 and *τ*_3 _= 0.52 are already fairly close to the freezing time in this case.

### (b) Complete model comparison with numerical method and experiments

The numerical–experimental work of Hindmarsh *et al*. [1] was selected for comparison with the complete model, more specifically for the following conditions: a droplet with radius *R* = 0.78 mm, ambient air temperature *T_∞_* = –19°C, nucleation temperature *T_n_* = −18.4°C and air flow velocity *v* = 0.42 m s^−1^. Hindmarsh *et al*. [1] measured the freezing time and the temperatures of the water droplets suspended by a thermocouple, then compared the experimental results with an approximate numerical analysis, considering a classical lumped model (which was termed the uniform temperature method in [1]), i.e. the droplet temperature is assumed to be spatially uniform during the cooling stages and constant in the freezing stage. A finite difference solution was also implemented for the cooling stages for verification of the lumped approach. The water thermophysical properties were assumed to remain constant and evaluated at the equilibrium freezing temperature (*T_f_*), equal to 0°C. The properties related to the air are evaluated at −19°C. Hindmarsh *et al*. [1] mention that the air currents had the humidity removed by silica gel, and thus we set *ρ_v_*_,∞ _= 0, which corresponds to assuming that the environment is of dry air. The heat and mass transfer coefficients (*h*) and (*h_m_*) were computed from the relations proposed by Beard & Pruppacher [41] for the Nusselt and Sherwood numbers. Hindmarsh *et al*. [1] neglected the heat conduction along the thermocouple and justified it through simulations. Table 3 presents the GITT convergence behaviour in terms of temperatures *T*(*r*, *t*) during the supercooling stage, for different values of *r* and *t*. Also shown is the convergence of the time for nucleation, in the last column. Convergence to at least five significant digits is achieved for truncation orders of *M* = 20 or higher. The temperature values within the droplet are rather uniform, in light of the fairly low values of Biot numbers, Bi*_c_*_,1 _= 0.1146, Bi*_m_*_,1 _= 0.0047 and Bi*_r_*_,1 _= 0.0012, explaining why the assumption of spatially uniform temperature used by Hindmarsh *et al*.[1] worked reasonably well in this case, as will be discussed in what follows.

**Table 3.** Convergence of droplet temperatures, *T*(*r*, *t*), in °C and time for nucleation, *t_n_*, in seconds, for different truncation orders *M*. Experiment of Hindmarsh *et al.* [1]. (*R* = 0.78 mm, *T_∞_* = −19°C, *T_n_* = −18.4°C, *v* = 0.42 m/s).

*M*	*T* (0, 5) (°C)	*T* (0, 20) (°C)	*T* (*R*/2, 5) (°C)	*T* (*R*/2, 20) (°C)]	*T* (*R*, 5) (°C)	*T* (*R*, 20) (°C)	*t_n_* (s)
10	−3.4910	−16.8407	−3.8240	−16.9129	−4.7968	−17.1250	27.9018
20	−3.4911	−16.8408	−3.8241	−16.9130	−4.7969	−17.1251	27.9017
25	−3.4911	−16.8408	−3.8241	−16.9130	−4.7969	−17.1251	27.9017
30	−3.4911	−16.8408	−3.8241	−16.9130	−4.7969	−17.1251	27.9017

For the present case (*R* = 0.78 mm, *T_∞_* = −19°C, *T_n_* = −18.4°C, *v* = 0.42 m s^−1^), the calculations of the recalescence stage result in *ϕ* = 0.7385 and *R*_ini_ = 0.758 mm. The solidification stage (stage 3) was solved for both hypotheses for the spatial distribution of the formed ice volume. The resulting times and droplet surface temperatures for stage 3 are enumerated in table 4, considering one value of time at the beginning and one near the end of the solidification period. The surface temperature for *t* = 5 s shows a very fast convergence to at least four significant digits with truncation orders as low as *M* = 10. For time values closer to the end of the stage 3, *t* = 23 s, convergence to six significant digits was achieved at higher orders, *M* = 60; however, convergence to four significant digits was achieved for truncation orders as low as *M* = 30. The results obtained from the two recalescence models were not markedly different, with the duration of the solidification stage for the first hypothesis being 24.11 s and 23.60 s for the other one. The duration of this stage experimentally measured by Hindmarsh *et al*. [1] was approximately 20 s.

**Table 4.** Convergence of freezing times and temperatures at the droplet surface for the solidification stage, considering two hypotheses for the spatial distribution of the formed ice after recalescence. Experiment of Hindmarsh *et al.* [1] (*R* = 0.78 mm, *T*_∞_ = −19°C, *T_n_* = −18.4°C, *v* = 0.42 m/s).

	recalescence model					
	ring of ice at droplet surface			uniform distribution of ice in droplet		
*M*	*t* (s)	*T*(*R*, 5) (°C)	*T*(*R*, 23) (°C)	*t* (s)	*T*(*R*, 5) (°C)	*T*(*R*, 23) (°C)
10	24.1065	−0.2131	−2.4746	23.5961	−0.0968	−2.8418
20	24.1093	−0.2131	−2.4731	23.5992	−0.0968	−2.8383
30	24.1102	−0.2131	−2.4726	23.6002	−0.0968	−2.8372
40	24.1106	−0.2131	−2.4724	23.6006	−0.0968	−2.8369
50	24.1109	−0.2131	−2.4722	23.6008	−0.0968	−2.8367
60	24.1111	−0.2131	−2.4721	23.6009	−0.0968	−2.8366

**Figure 3.**
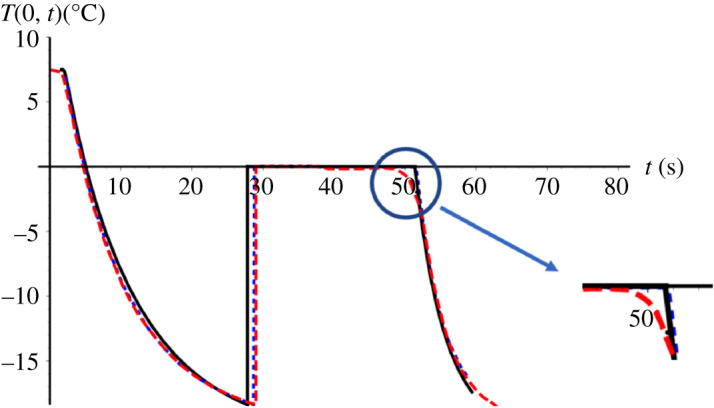
Comparison of GITT, classical lumped approach [1] and experimental results [1] for the droplet centre temperature, *T*(0,*t*), along the entire process: GITT (*M* = 60) (black solid line), classical lumped approach (dotted blue line) and experimental analysis (dashed red line) [1]. *R* = 0.78 mm, *T_∞_* = −19°C, *T_n_* = −18.4°C, *v* = 0.42 m s^−1^. (Online version in colour.)

Figure 3 shows a comparison between the GITT solution, the classical lumped approach proposed by Hindmarsh *et al*. [1] and the experimental temperature evolutions at the centre of the droplet for the entire process, including all four stages. For the solidification stage, the hypothesis that considers a homogeneous distribution of the ice formed during the recalescence period has been adopted. A truncation order of *M* = 60 was employed in this comparison. An excellent overall agreement between theoretical predictions and experimental measurements is achieved. The deviations between the GITT solution (solid black line) and the classical lumped approach (dashed blue line) by Hindmarsh *et al*. [1] can be explained through the distinct modelling adopted. Hindmarsh *et al*. [1] model assume uniform temperature within the droplet, both for the supercooling stage and the freezing stage. In fact, Hindmarsh *et al*. [1] themselves also compared these two modelling approaches, distributed and lumped, pointing out deviations in the results obtained.

For additional assessment of the GITT results, a purely numerical analysis of the first stage, considering the full heat conduction equation for distributed parameters, was performed here using the *NDSolve* routine from the *Mathematica* system v. 11.3 [42]. The *NDSolve* algorithm implements the method of lines for the solution of partial differential equations, with variable mesh and variable difference order. This automatic solver for handling partial differential equations is the same employed in solving the nonlinear transformed ODE system, but is here also adopted in verifying the GITT solution for the first stage. The agreement between the GITT and the methods of lines numerical solution is excellent, presenting a relative deviation of around 0.07% for the duration of the supercooling stage. For improved agreement with the fully converged GITT solution, a very refined mesh is required by *NDSolve* through the corresponding prescribed input parameters. Excellent agreement was also observed between the GITT solution and the numerical solution via *NDSolve* for the cooling (fourth) stage, with a relative deviation of around 0.09% for the time calculated from the beginning of the fourth stage to the time when the droplet centre reaches the ambient air temperature (*T_∞_*). The comparison between GITT and *NDSolve* was not possible for the freezing (third) stage, due to the moving boundary, which limits the use of the general-purpose routine.

The fact that the experimental analysis shows a slightly longer cooling period in comparison with both theoretical solutions, as noted in the first branch of figure 3, could be explained due to the presence of dissolved air inside the liquid droplet, which leads to variations on thermal conductivity and capacitance, thus the slightly longer cooling time detected in the supercooling stage. Also, a slightly shorter freezing period and less abrupt transition to the cooling period is observed in the experimental results with respect to the theoretical ones, as can be clearly observed in the zoomed in portion of figure 3. The thermocouple measuring location uncertainty may explain this deviation, since one cannot ensure that the thermocouple is positioned precisely at the droplet centre [22].

### (c) Parametric analysis

A parametric analysis is undertaken next using the same validation case above as a reference to analyse the influence of different quantities and parameters. First, the relative importance of the three modes of energy transfer at the droplet surface is examined. The heat fluxes related to convective heat transfer (*q_c_*), radiative heat transfer (*q_r_*) and convective mass transfer (*q_m_*) are evaluated from:

4.1 $$\begin{array}{rl} q_{c} & =h(T(R,t)-T_{\infty }), \end{array}$$

4.2 $$\begin{array}{rl} q_{r} & =\varepsilon \sigma ({\left(T(R,T)\right)}^{4}-{T_{\infty }}^{4}) \end{array}$$

4.3 $$\begin{array}{rl} \mathrm{and}\;\;q_{m} & =h_{m}L_{e}(\rho_{v,l}(T(R,t))-\rho_{v,\infty }). \end{array}$$

**Figure 4.**
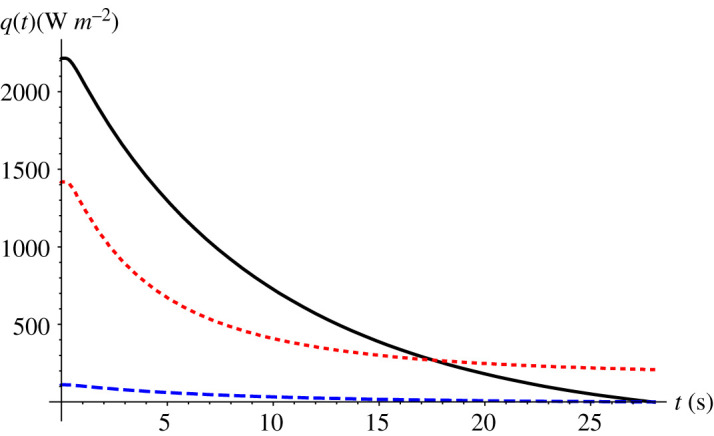
Evolution of the three heat flux components, *q*(*t*), for convection (solid black line), convective mass transfer (dotted red line), and radiation (dashed blue line) in the supercooling stage. Data from Hindmarsh *et al*. [1]: *R* = 0.78 mm, *T_∞_* = −19°C, *T_n_* = −18.4°C, *v* = 0.42 m s^−1^. (Online version in colour.)

Figure 4 represents the evolution of all three heat fluxes during the supercooling stage, as computed from the GITT formalism. The overall contribution of each heat transfer mode to the heat exchange between the droplet and external environment at stage 1 can be obtained by integrating equations ((4.1)–(4.3)) between the limits *t* = 0 and *t* = *t_n_*, where *t_n_* is the nucleation time (i.e. the duration of the supercooling period herein). As expected, convective heat transfer accounts for most of the heat exchange with the surroundings, around 58.2%, while the convective mass transfer accounts for 39% of the overall heat exchange, and radiative heat transfer for only 2.8% of this total. From figure 4, it can be noted that, after around 19 s, the convective mass transfer becomes dominant. As the droplet surface temperature approaches the external ambient temperature, the difference (*T*(*R*, *t*) − *T*_∞_) approaches zero, together with the convective heat flux, while the difference (*ρ_v_*_,*l*_(*T*(*R*, *t*)) − *ρ_v_*_,∞_) present in the heat flux related to convective mass transfer approaches a fixed value, since *ρ_v_*_,∞ _= 0. The importance of convective mass transfer at low-temperature differences had already been pointed out by Strub *et al*. [43], who observed stabilization temperatures lower than the ambient ones.

**Figure 5.**
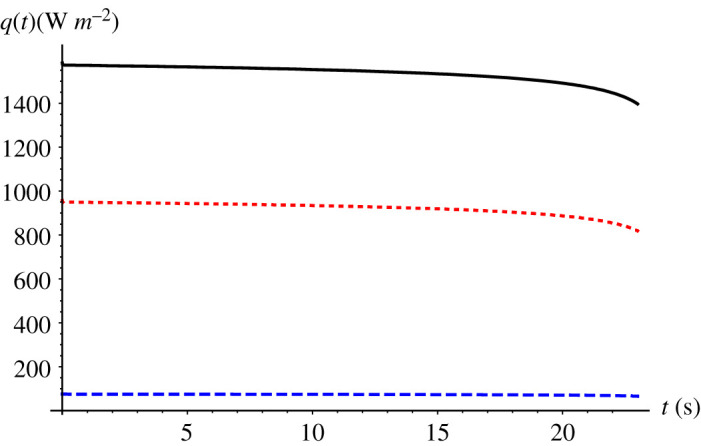
Evolution of the three heat flux components, *q*(*t*), due to convection (solid black line), convective mass transfer (dotted red line) and radiation (dashed blue line) along the solidification stage. Data from Hindmarsh *et al*. [1]: *R* = 0.78 mm, *T_∞_* = −19°C, *T_n_* = −18.4°C, *v* = 0.42 m s^−1^. (Online version in colour.)

Figure 5 again shows the evolution of the three modes of heat fluxes but now for the solidification stage. In this case we need to substitute the latent heat of evaporation (*L_e_*) and *ρ_v_*_,_*_l_* for the latent heat of sublimation (*L*_sb_) and *ρ_v_*_,_*_s_* in equation (4.3). The convective heat transfer remains dominant, accounting for 61% of the overall heat exchange in this period, while convective mass transfer and radiative heat transfer roughly account for 36% and 3%, respectively. Here the convective heat transfer has a higher participation in the overall heat exchange compared with the supercooling stage, because the droplet surface temperature remains practically constant during this stage, but different values of the Biot number did lead to modification of this behaviour, as will be observed in what follows.

Quantifying the pathways of heat dissipation, as in figures 4 and 5, is important in assessing the role of each transport mechanism in the freezing process and its influence on the freezing time. Castillo *et al*. [44] performed a similar theoretical evaluation for heat dissipation rates for the freezing of a droplet resting on a cooled substrate. They estimated the fraction of heat that is lost through the substrate and through the droplet/air interface, either by natural convection or by convective mass transfer, and their analysis has motivated the present comparison. Looking just at the droplet/air interface, the chosen parameters were the ratio between the convective heat flux and total heat flux, and the ratio between the heat flux related to convective mass transfer and total heat flux, both ratios calculated at the droplet/air interface and at the beginning of the freezing stage. These ratios were approximately 0.68 and 0.30, respectively, for the 10.1 µl droplet studied by Castillo *et al*. [44]. In the present work, these same ratios were nearly 0.60 and 0.36, respectively, for a 2 µl droplet. Disregarding the different boundary conditions and volumes analysed in the two studies, the ratios show plausible orders of magnitude, even more so when taking into account the fact that in the present work the air surrounding the droplet was assumed dry, increasing the influence of the convective mass transfer.

The influence of the Stefan number and the other dimensionless parameters (Bi*_c_*_,3_, Bi*_m_*_,3_ and Bi*_r_*_,3_) in the droplet cooling process was also analysed. The Stefan number (*St*) is a frequently encountered dimensionless parameter in phase change problems, being interpreted as the ratio of sensible and latent heats of the system. In the present parametric study, the external air temperature was changed in order to yield different values of the Stefan number. Table 5 summarizes the cases analysed. Case 1 corresponds to the experiments of Hindmarsh *et al*. [1], used in the above validation. For Case 1, the Stefan number is 0.11 and the freezing time is computed as 23.6 s.

**Table 5.** Test cases for analysing the influence of Stefan number and Biot numbers.

parameters	Case 1 (validation)	Case 2	Case 3
*T*_∞_ (**°**C)	−19	−40	−19
*St*	0.11	0.24	0.11
Bi***_c_***_,3_	0.0347	0.0345	0.0639
Bi***_r_***_,3_	0.0004	0.0003	0.0004
Bi*_m_*_,3_	0.0212	0.0095	0.0389
*v* (m s^−1^)	0.42	0.42	2.0

**Figure 6.**
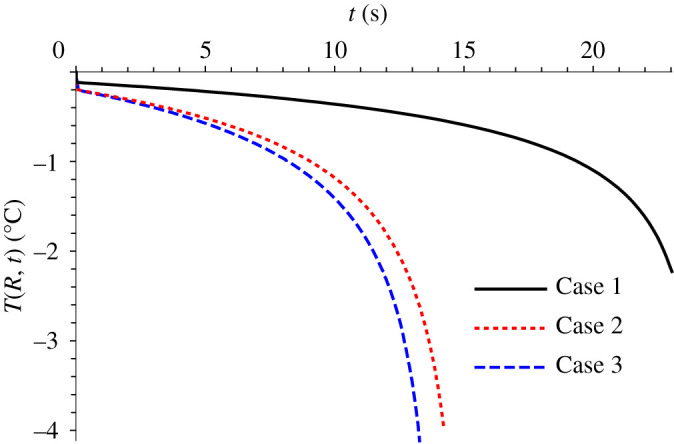
Evolution of droplet surface temperature, *T*(*R*, *t*), along the solidification stage for the three cases of parameters variation in table 5.

Figure 6 shows that the droplet surface temperature, *T*(*R*, *t*), varies little along the solidification stage for this case. In addition to enlighten the weight of different heat transfer modes in the freezing process, as mentioned earlier, the droplet surface temperature is an important indication of the temperature gradient in the solid phase within the droplet, since the freezing front temperature remains at the equilibrium freezing temperature (*T_f_*), i.e. 0°C. For Case 1, 22.5 s after the start of solidification, the surface temperature drops by only 2.5°C. Towards the end of the solidification period, the droplet surface temperature varies more markedly due to the reduction in the latent heat released at the freezing front.

In Case 2, the Stefan number is about 2 times higher than in the base case, after changing the external air temperature from −19°C to −40°C. In this case, the freezing time is approximately 14.7 s. From table 5 it can be observed that the values of the three parameters, Bi*_c_*_,3_, Bi*_m_*_,3_ and Bi_r,3_, also reduced with the change in the ambient temperature. The parameter Bi*_c_*_,3_ was the least affected one and, as a consequence, the convective heat flux contribution jumped from roughly 60% in Case 1 to around 77% in Case 2. The direct comparison of *T*(*R*, *t*) in Case 1 and Case 2 from figure 6 also shows that the droplet surface temperature variation is pronounced in Case 2. Around 14 s after the onset of solidification, the surface temperature has already dropped by almost 4°C. The increase in Stefan number represents a relative increase in sensible heat transfer in comparison with the latent heat, therefore, one may expect a more significant variation of temperatures during the phase change process. This observation is crucial for processes in which the temperature gradients have to be controlled, such as in the pharmaceutical, food and metallurgical industries, because the constitution of the formed solid is affected by the magnitude of this gradient. In Case 3, the values of Bi*_c_*_,3_, Bi*_m_*_,3_ and Bi*_r_*_,3_ were increased with respect to Case 1, for instance through changes in the air flow velocity, but keeping the Stefan number equal to Case 1. By changing the air flow velocity from 0.42 to 2 m s^−1^, Bi*_c_*_,3_ and Bi*_m_*_,3_ values almost doubled in comparison with Case 1, although the value for Bi*_r_*_,3_ remained the same. The freezing time in this case was around 13.8 s, and figure 6 again shows the evolution of the droplet surface temperature along the solidification. The decrease in the droplet surface temperature in Case 3 is a little bit faster than in Case 2, i.e. in just 13 s after the start of solidification, the droplet surface temperature has already reduced by almost 4°C, illustrating that the changes in the Biot numbers have a more significant influence compared with the influence of the Stefan number on the temperature gradients along the phase change process.

With respect to the problem modelling, for sufficiently mild temperature gradients within the medium, such as in Case 1, an improved lumped-differential approach can be recalled that markedly simplifies the formulation with an acceptable loss in precision, as thoroughly discussed in [31]. Then, the original partial differential equations can be reduced to ordinary differential equations by the reformulation process.

**Figure 7.**
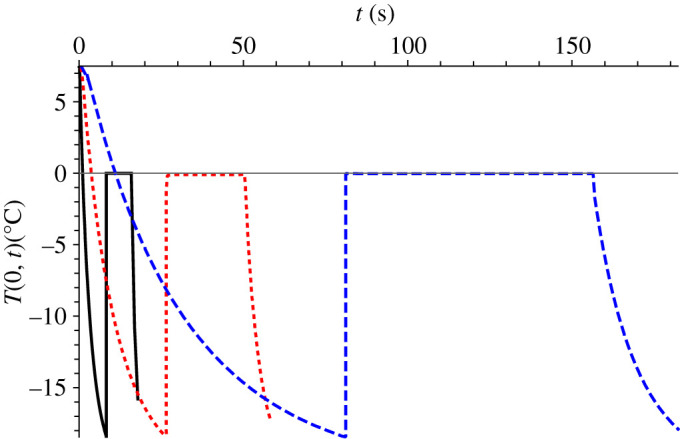
Evolution of droplet centre temperature, *T*(0*, t*), base Case 1 but for three different droplet radius: 0.39 mm (solid black line), 0.78 mm (dotted red line) and 1.56 mm (dashed blue line). *T_∞_* = −19°C, *T_n_* = −18.4°C, *v* = 0.42 m s^−1^. (Online version in colour.)

Figure 7 shows the droplet centre temperature, *T*(0, *t*), as a function of time for the base Case 1 but with three different droplet radius: (i) 0.39 mm, (ii) 0.78 mm and (iii) 1.56 mm. As expected, the larger the droplet, the greater is the freezing time, being roughly 18.7 s for the 0.39 mm radius and 183.4 s for the 1.56 mm radius, i.e. a 10 times rise for an increase of 4 times in the droplet radius. It has also been observed that with the increase in the droplet size, the contribution of the radiative heat flux became more significant in all the stages. In the supercooling stage for example, it increased from 1.7% to 2.8% when the droplet radius was doubled from 0.39 to 0.78 mm and to 4.1% when the radius was again doubled to 1.56 mm. In this case, the contribution of the convective heat flux and convective mass transfer experienced a mild decrease with the radius increase. The rise of the radiative heat flux participation is explained because of the influence of the droplet radius in the heat and mass transfer coefficients (*h*) and (*h_m_*), since the value of both coefficients decreases with the increase in the droplet radius.

## 5. Conclusion

Freezing of supercooled liquid droplets suspended in air is modelled through a transient one-dimensional heat conduction formulation in spherical coordinates that accounts for the nonlinear effects of convective heat transfer, convective mass transfer and thermal radiation at the droplet surface. The freezing process is divided into four distinct stages, namely, supercooling, recalescence, freezing and cooling stages. Except for the recalescence stage, which is represented by a simplified model as an instantaneous nucleation process, the other three stages result in nonlinear partial differential equations that are solved by the GITT, including the moving boundary problem that represents the freezing stage. This hybrid numerical–analytical approach with automatic accuracy control is here employed based on a nonlinear eigenvalue problem for representing the eigenfunction expansions. The original nonlinear boundary conditions, after an implicit filtering to eliminate the associated source terms, are directly accounted for by the chosen eigenfunctions and eigenvalues. This approach results in a marked improvement on convergence rates for the desired potentials, including the temperatures at the interface and the moving interface position. The model and solution methodology are both verified with existing approximate analytical and numerical solutions for simpler formulations and validated against experimental and numerical results in the literature. Then, parametric combinations are chosen to analyse some of the relevant physical aspects in determining the freezing time, surface temperature and the evolution of the freezing front. The present approach does not introduce any approximations for the spatial dependence of the temperature field, as required in lumped system approaches, or any limitations on the magnitude of the Stefan number, which is typical in perturbation-type approaches. In addition, it can also be extended to the two-dimensional formulation in non-spherical geometries required to handle the situation of a droplet in direct contact with a solid surface, by considering the related two-dimensional eigenvalue problems.

## Data accessibility

The Mathematica software codes employed in generating the figures and tables are readily available from the UCL research data repository at the link below: Carvalho, Igor; Cotta, Renano; Naveira-Cotta, Carolina; Tiwari, Manish (2021): CODES FOR "HYBRID INTEGRAL TRANSFORM ANALYSIS OF SUPERCOOLED DROPLETS SOLIDIFICATION". University College London. Software. https://doi.org/10.5522/04/14165063.v1.
